# Challenges and opportunities of psychological aging research

**DOI:** 10.1007/s10433-025-00891-9

**Published:** 2025-11-04

**Authors:** Paolo Ghisletta

**Affiliations:** Department of Psychology, Faculty of Psychology and Educational Sciences, University of Geneva, Boulevard du Pont d’Arve 40, 1211 Geneva, Switzerland

**Keywords:** Psychological aging, Lifespan development, Artificial intelligence in research

## Abstract

The scientific study of psychological aging is very challenging due to the complex, multidimensional, multi-directional, and highly variable nature of change processes observed in adulthood and old age. Psychological aging encompasses phenomena that require interdisciplinary efforts to be understood. Recent advancements in technology (e.g., wearable devices, apps offering digital metrics, multimodal data collection, artificial intelligence, and big data algorithms) reconfigure the epistemology of behavioral and social sciences but can, if properly applied and analyzed, enhance our understanding of the mechanisms behind age-related within-person change. As researchers in the field of psychological aging, we must continually train in various domains and keep abreast of new methodologies, with the aim of advancing theoretical perspectives on aging. Collaborative, open, and cumulative research efforts are key to developing our knowledge of psychological aging. Additionally, as privileged observers of aging mechanisms and processes, we bear the responsibility to challenge stereotypes surrounding aging and help educate professionals in related fields who work with older populations. There is also a critical need for accurate scientific information about psychological aging to inform public and social policies, making our contributions even more valuable. This paper explores some of these challenges of psychological aging research and the opportunities they present.

## Introduction

The World population has been rapidly aging, largely due to medical, health care, and technological progress leading to increased life expectancy (United-Nations 2024). This shift in demographic age composition implies that specific psychological, health, social, and societal issues relevant to older people gain dramatically in importance and are in dire need of educated, science-based evidence. This paper offers a critical examination of research in the field of psychological aging, by first reviewing specific challenges of this broad discipline, followed by a discussion of opportunities that can enrich how psychological aging research is carried out, and finally attempts at enumerating several broader concerns that researchers in this field can shape. As any review cannot be fully comprehensive, the readers may find themselves in agreement with some points, while in discord with others. Either way, it is hoped that this review may at least offer some fresh perspective and stimulate further reflection on psychological aging research.

## Challenges

The field of psychological aging research is allegedly arduous. First, it suffices to read the table of contents of any dedicated textbook or special issue to appreciate the variety of themes subsumed by this broad field. Aspects commonly discussed in the realm of psychology of aging include genetic, biological, health, physical, cognitive, educational, personality, emotional and affective, motivational, religious and spiritual, technological, work and retirement, narrow and broad social, and societal aspects (Bjorklund and Earles 2021; Cavanaugh and Blanchard-Fields 2018; Poulton et al. 2023; Rolison and Freund 2024; Schaie and Willis 2016; Whitbourne 2001). Given the interdependencies of these life spheres within an individual across the entire lifespan and the predictive power of childhood individual differences for old-age characteristics, it is illusory to conceptualize any one of them in total isolation, without at least the recognition, if not direct empirical assessment, of its associations with others (Baltes 1997). Moreover, the cumulative nature of life-long experiences enjoyed and endured by older individuals increases the complexity of their systems in biological aspects (Cohen et al. 2022) but also in psychological, cognitive, social, and physical functioning (Kok et al. 2025), compared to humans in previous stages of their life.

### The lifespan approach

Most scholars of the psychology of aging embrace the lifespan paradigm put forth in the 1970s, most notably after the West Virginia University Conference on Life-Span Developmental Psychology held in 1969 (Goulet and Baltes 1970). Among the key principles of lifespan psychology are the 3 Ms: Multidimensionality, multidirectionality, and multifunctionality, which are intertwined concepts that define development as a perpetual dynamic system shaped by gains and losses (Baltes 1987). Multidimensionality refers to the facets of plurality during development, such that ontogeny is not constrained to a single criterion. The classical example is that of intelligence, which is not a single entity but rather a system of multiple subcomponents (e.g., speed, vocabulary, memory, fluency, reasoning). Multidirectionality, relatedly, speaks to the diversity of ontogenetic dynamics followed by interrelated subcomponents of a given developmental entity (e.g., speed tends to decline already in early adulthood, whereas vocabulary tends to remain stable, or even increase, across most adulthood; Ghisletta et al. 2012; Singer et al. 2003). Finally, multifunctionality speaks to the different roles that a developmental component can play during the lifespan (e.g., cognitive strategies that are functionally related to successful task completion at one point and thus can be applied to solve a given task of lower complexity, can lead to failure in more advanced tasks, when the greater complexity of the task requires refining, if not replacing, old strategies; Baltes et al. 2006). At times, multicausality is also mentioned, meaning that the same developmental expression may have been reached via a multitude of routes, and multiple causes (e.g., genetic, epigenetic, contextual, environmental) may precipitate similar processes leading to the same outcome (Baltes et al. 2006).

Another basic tenet of lifespan developmental psychology is the need for interdisciplinarity. Individuals’ developmental paths are embedded within constantly fluctuating contextual realities (Elder and Johnson 2003; Settersten et al. 2024) and are prone to biology-based influences, although to varying degrees across different domains and stages of life (Baltes 1987). Nevertheless, the constructivist perspective of lifespan development assumes that human development is built upon the intricate interactions among biological, psychological, and social drives to which individuals contribute agentically. It follows that some knowledge of related disciplines (e.g., genetics, medicine, neuroscience, sociology, demography, epidemiology, economics, anthropology, geography) is needed to better apprehend the convoluted nature of the complex interplays that ultimately shape human development (Blackburn 1991; Cabeza et al. 2017; Deary et al. 2010; Levy et al. 2005). For a psychological aging scholar, this entails embracing a large perspective on one’s precise object of inquiry, thereby calling for at least more comprehensive theoretical literature reviews, if not full-blown interdisciplinary studies, necessarily in a collaborative spirit to maximize the pool of expertise available (Deary et al. 2021; Gow et al. 2007; Knight 2012; Kornadt et al. 2020). Animal models of aging processes can also be highly informative for understanding human development (Kenyon et al. 1993; Phillips and Roth 2019; Rothwell et al. 2021). In sum, psychological aging research needs to enrich its perspective by integrating knowledge from related disciplines, thereby going beyond multidisciplinary efforts to transcend disciplinal boundaries in the aim of creating a unified approach.

### Increased variability

The seminal article by Nelson and Dannefer (1992) examined the study of interindividual variability in gerontological and child developmental research. While many authors theorized about the high degrees of, if not an increase in, variability among older people, few documented this phenomenon empirically, and characterization in terms of central tendency measures remained, paradoxically, predominant. Yet, in their literature review they found that 43% (*n* = 54) of the 127 retained gerontological studies reported measures of variability, whereas this proportion was 24% (*n* = 14) in the 58 child-development studies retained. Furthermore, 65% of the gerontological studies considering variability indices witnessed an increase in heterogeneity, whereas in child studies fewer did (50%). This corroborates the greater amount of variability reported in studies on aging compared to child development, leading some to state that older adults appear more different from each other than do younger adults (Rolison and Freund 2024). In medicine, too, it is well recognized that older adults have greater clinical and biological heterogeneity than younger adults (Austin et al. 2024). Similarly, intraindividual variability (short-term, reversible within-person change; Nesselroade 1991) in older adults is also greater than that of younger adults, and often comparable to (if not greater than) that of children (Diehl et al. 2015; Dykiert et al. 2012; Ram et al. 2009). This observation holds across many behavioral outcomes and is bolstered by much neuroscientific evidence (MacDonald et al. 2006; Lövdén et al. 2013).

This reality makes it all the more arduous to conclude about generalizations (in terms of laws, theories, hypotheses, or even simple observations) dictating the processes of psychological, but also biological, physical, and social aging. Psychological aging researchers must thus be equipped with tools capable of apprehending different types of variability and possibly further elucidate theories and hypotheses about aging. Furthermore, given that older people grow not just in number but also in diversity, aging research needs to strive more intently at studying diversified populations in terms of race or ethnicity, socio-economic status, immigration status, family structures, gender identification, sexual orientation and more, by, for instance, adopting more refined sampling schemes (Diehl et al. 2020; Karel et al. 2012; Meeks et al. 2021; Zacher 2025). Although often within-group (between-person) variability is greater than between-group differences, conclusions about interindividual and intraindividual variability estimated from a given set of sample characteristics may not generalize to other samples (Deater-Deckard et al., 2018).

### Methodological confounds

The common definition of psychological aging as the field that studies how people’s psychological characteristics change with age camouflages many factors intertwined with each other that may cloud attempts at achieving this goal (Salthouse 2000). First, while chronological age remains a convenient carrier variable whose increase covaries more or less strongly with many processes of different nature, it remains the simple cumulation of time since birth (i.e., date of assessment minus date of birth), thus void of any psychological interest in itself (Wohlwill 1973). Epistemologically, age does not permit understanding processes that evolve as we distance ourselves from birth; it simply provides a time basis to order these processes. Attempts at elucidating correlates of age effects include estimating one’s functional age (via biomarkers such as sensorimotor, anthropometric, physiological, and dental variables; Anstey et al. 1996), describing processes as a function of time to a specific event (such as hospitalization, Wilson et al. 2012, preclinical dementia diagnosis, Sliwinski et al. 2003, or death, Gerstorf et al. 2013), and conditioning age-related changes to events (as in joint longitudinal–survival models, Ghisletta 2008; Ghisletta et al. 2025; McArdle et al. 2005). These attempts may not conclusively unravel the nature of age effects, but they often force scholars to excogitate what age-related processes may influence the process under investigation.

A seminal paper by Schaie (1965) elucidates the distinction between age, time of measurement, and birth cohort effects on developmental processes. Cross-sectional (single measurement of different birth cohorts at a given time point), longitudinal (repeated assessments of a given birth cohort at different time points), and time lag (multiple assessments of different birth cohorts at different time points, such that all cohorts are assessed at the same age) designs necessarily confound two of these sources of variation while keeping the third constant. In cross-sectional studies, age and birth cohorts are confounded, whereas time of measurement is constant; in longitudinal studies, age and time of measurement are confounded, whereas birth cohort is fixed; finally, in time-lag studies, age is fixed, whereas birth cohorts and time of measurement are confounded. Sequential-type designs, where at least two such studies are carried out in parallel, allow partially disentangling the three developmental effects. The most frequent is the so-called cross-sequential (a.k.a. accelerated) design, in which multiple birth cohorts are assessed at multiple times of assessment, corresponding to multiple simultaneous longitudinal studies of different birth cohorts (Bell 1953). These require, nevertheless, testing whether the longitudinal sequences differ across the cohorts (Cáncer et al. 2023). Note that in recent years, there has been a resurgence in interest in estimating cohort effects on various psychological aging phenomena (e.g., Drewelies et al. 2019).

One of the dominant methodological beliefs in lifespan research is that longitudinal studies are necessary to infer about intraindividual (within-person) change (Baltes and Nesselroade 1979). Any deviation from this truism would entail taking shortcuts implying that interindividual (between-person) differences estimated from cross-sectional studies would inform about how individuals change (Raz and Lindenberger 2011). Yet, longitudinal studies are not without caveats (Campbell and Stanley 1963; Salthouse 2000). Prime among these are retest (or practice) effects, which can be estimated, under given assumptions, either methodologically (by comparing different samples equivalent in age structure but with varying amounts of previous test exposure, Schaie 1965) or statistically (by testing, in the same model, an age-based predictor against another predictor accounting for previous test exposures, Rabbitt et al. 2001).

Another possible drawback of longitudinal studies is selective attrition, which entails that longitudinal samples become increasingly positively biased at each additional wave of assessment, thereby reducing their representativeness of the initial intended population (Baltes et al. 1971), whether for mortality or experimental reasons (Lindenberger et al. 2002). Ongoing participants in longitudinal studies are often endowed with higher cognitive performance, more agreeable and open personality, better physical and perceived health, and generally better socio-economic status (Ghisletta and Spini 2004; Salthouse 2014). Selective attrition may compound with selective survival effects, which bias estimates already in cross-sectional studies if health factors influencing participation differ with age (i.e., survivorship bias; Wald 1943). Luckily, in longitudinal studies these effects can be reduced by adding new (random and representative) refreshment samples at successive waves (Kish and Hess 1959). Care must thus be taken in the analyses to correct for estimation bias due to selectivity, possibly by identifying and including variables probabilistically associated with the dropout process (Graham 2003) or employing modern statistical methods, such as multiple imputation or factored regression specifications (Enders 2025). Contingent upon careful treatment and analyses, longitudinal studies remain invaluable sources of within-person information, even with substantial attrition rates (Gustavson et al. 2012).

Finally, it is not uncommon that in long-lasting longitudinal studies (such as nation-wide panels) researchers decide to replace an old instrument assessing a given construct with a newer one (Ruspini 2003). This implies that longitudinal measurement equivalence is no longer guaranteed, as assessments are based on different scales of measurement, rendering difficult the estimation of models analyzing old and new instruments. Classical methods to handle changes in instrumentation include standardization techniques, sometimes based on norms, but which lose information about systematic change in the means and variances and fail if old and new scores do not correlate highly. Modern methods allow calibrating and linking different measurements of the same construct by capitalizing on item response theory merged with models about change, as long as some participants were assessed with multiple instruments (McArdle et al. 2009). Similarly promising techniques facilitate planning for scale alterations owing to substantive reasons (e.g., age appropriateness of measurements).

### Open science

The replicability crisis in science may have harmed not only the reputation of the professions themselves, but also the level of confidence by the lay public in scientific discoveries (especially in psychology and social sciences, when results often go against common sense; Bertenthal 2002). Aging research has perhaps been slower than other fields in adopting open-science standards (Pruchno et al. 2015), partially owing to specific features that complicate the creation of a general policy for open-science practices (Stine-Morrow 2022). For instance, failure to replicate may mirror that some findings do not hold across different birth cohorts or sociocultural groups. When findings are generated via secondary analyses on numerous variables from large datasets, a myriad of minor decisions need to be implemented (about variable coding, sample considerations, analytical strategies, etc.). These issues notwithstanding, thanks to new research practices and technological tools, concrete means are now available to implement open-science approaches also in aging research (Isaacowitz and Lind 2019; Pruchno et al. 2015; Stine-Morrow 2022).

## Opportunities

Though performing research in psychological aging is challenging, there are various reasons as to why scientific knowledge from this broad discipline is urgently needed. Additionally, recent technological advances offer a host of opportunities to improve how we carry out research to elucidate psychological aging phenomena and mechanisms.

### Global demographic changes

Demographers have long alerted us of the global aging of the World population. Western countries that experienced the baby boomers generation (i.e., increased fertility rates after World War II, between 1946 and 1964) have also been facing important increases in life expectancy (Pew-Research-Center 2018). At the same time, since 1990, infant mortality has been decreasing in most countries worldwide (United-Nations 2024). Together, such factors contribute to global demographic changes resulting in pyramids whose large bases (representing young children) are narrowing while their thin tips (depicting older adults) are widening, such that the pyramids are slowly mutating to domes (Parker 2014). Current United Nations projections are that individuals aged 65 years or older will increase from 761 M (1 in 11) in 2021 to 1.6B (1 in 6) by 2050, and those aged 80 years or more will triple in number by 2050 (United-Nations 2024).

These dramatic demographic changes imply that expertise about aging, at many different levels, is more than ever before needed. Bearing in mind the increased variability in psychological functioning observed in older adults, the distinction between the positive views of the third age (the young old), often paralleled to successful aging, and the vulnerabilities and reduced optimization of the fourth age (the old old) is extremely important, both for applied settings and social policies (Baltes and Smith 2003). Although the majority of individuals in the third age are overall in good health (cognitively, socially, and physically, Williamson and Christie 2009), those suffering from mental health issues are mainly affected by anxiety, mood, impulse-control, and substance use disorders, often in comorbidity (Karel et al. 2012). Very old adults, on the other hand, are more likely to develop neurodegenerative diseases (e.g., Alzheimer’s disease or other forms of dementia, Parkinson’s disease), making detection of early pathological cognitive decline a research priority (Harrell et al. 2024; Muñoz 2021). Inevitably, health practitioners will care for more and more old and very old adult individuals, yet not nearly enough have been trained appropriately, to the point that some talk about the “upcoming crisis in geriatric mental health care” (Holtzer et al. 2012). It was estimated that in 1999 there were about 3000 psychiatrists and psychologists in the USA specialized in working with older adults, but that at least three times as many were needed to meet the demands (Jeste et al. 1999). This crisis impinges also on medical generalists, who often represent the first level of care for patients, but for whom, paradoxically, training in aging and gerontopsychology has not necessarily increased and is lagging behind (Holtzer et al. 2012). Finally, the recent COVID-19 pandemic (caused by acute respiratory syndrome coronavirus 2, SARS-CoV-2) had major consequences not only on younger, but also on many older people’s quality of life following the physical distancing strategies implemented by many governments. Besides the resurgence of ageism attitudes and behaviors (Ayalon et al. 2021), social isolationist measures were experienced negatively by many older adults in terms of general mental health (Hwang et al. 2020), which could lead to increasing first rates of anxiety or depression in future years (Carpenter et al. 2022). Thus, the urgency to infuse specific knowledge about older adults in various health-related professions is stronger than ever.

Although neural deterioration occurs normatively in older adults (Raz 2000), the potential for neuroplasticity remains and can be fostered by appropriate training and intervention programs (Lövdén et al. 2010; Park and Bischof 2013). Multiple studies have shown the efficacy of cognitive training on both behavioral (e.g., increased performance, Park et al. 2013) and neural (e.g., increased connectivity, volume, thickness, and density, Deng et al. 2019; Nguyen et al. 2019) features. At times, such training programs proved beneficial also on non-trained aspects, such as on instrumental activities of daily living (Rebok et al. 2014). Overall, then, efficient training and intervention programs may, ultimately, reduce or delay cognitive decline in older adults. Similarly, intervention trials aimed at more general lifestyle changes (e.g., weight loss, increased exercise, especially aerobic) have shown efficacy in terms of improved physical functioning and biomarkers of physical frailty in older adults (Porter Starr et al. 2014) but also cognitive functioning (Hertzog et al. 2008). Given that the most recent broad consensus statement on the prevention of dementia identified 14 (health, physical, social, and psychological) risk factors that are largely lifestyle-related, and thus potentially modifiable (Livingston et al. 2024), there is a compelling need to design, evaluate, and promote intervention programs aimed especially at older adults (Carpenter et al. 2022; Harrell et al. 2024). But such programs ought to be especially tailored to the needs and capacities of older adults and possibly individualized, which requires, again, specific science-based knowledge about old age (Livingston et al. 2024). Recently, it has also been proposed that the emergent field of precision medicine within geriatrics (i.e., augmenting medical treatments by including not only individual genetic and protein information but also social, demographic, behavioral, and clinical characteristics; Austin et al. 2024) be applied to understand, prevent, and treat age-related cognitive impairment (Ryan et al. 2019).

### Technological advances

The COVID-19 pandemic put a halt to many research projects owing to the social and physical distancing strategies implemented to limit the spread of the virus. Worldwide, governments suggested or enforced isolation measures, thereby pressing aging researchers to switch their data-collection methods from physical presence to distance modes of various types. Methods such as phone-assisted personal interviewing (PAPI), computer-assisted telephone interviewing (CATI), and computer-assisted web interviewing (CAWI), which have been used for decades, often became the only choice available to researchers who quickly had to adapt to pursue their research endeavors. Notable examples include national or international large-scale panels such as the National Social Life, Health, and Aging Project (NSHAP, Hanis-Martin and Schwartzman 2025), the Health and Retirement Study (HRS, Smith et al. 2023), and the Survey of Health, Ageing, and Retirement in Europe (SHARE, Scherpenzeel et al. 2020). Some studies also developed kits that were sent to participants to collect sensory and various other biomarkers (salivary and blood samples) (e.g., the BioBox, Wiencrot et al. 2025). Those who enjoyed data collected both in presence and remotely within the same waves of assessment, and thus were able to compare the quantity and quality of responses, often concluded that response rates were greater in the distant modes (Wiencrot et al. 2025) and that, if quantitative differences emerged, they tended to be rather inconsequential (Kliegel et al. 2007; Smith et al. 2023). This conclusion also appears to hold when collecting online reaction times (although minor differences may emerge across different platforms, browsers, or operating systems; Anwyl-Irvine et al. 2021; Semmelmann1 and Weigelt 2017). It thus appears that if design and, consequently, analytic strategies are carefully attended to, difficulties such as reduced possibilities to clarify questions, the need for some minimal access to and use of technological devices, and possible underrepresentation of individuals with low levels of literacy can be overcome to obtain robust remote measurements (Hensen et al. 2021; Pudelek et al. 2025). Yet, we must remain wary as online data collection does not guarantee that participants are following instructions, such as not writing words to be retained in a recall task (Smith et al. 2023).

The use of wearable devices and apps capturing digital metrics has the potential, if properly presented, explained, and accompanied, to measure more validly the general functioning of older adults’ and to support health intervention programs for them to potentially ameliorate their health (Lyons et al. 2017). Yet, few (usually smaller scale and relatively short) studies have evaluated the use of wearable devices for health intervention (Coughlin and Stewart 2016). In older adults, awareness of the wearable devices’ potential for early detection and prevention of complications and emergencies should be raised (Kekade et al. 2018). Among the factors influencing the use of wearable devices are both intrinsic and extrinsic motivational factors, the perceived complexity of the device, and the capacity of the device to fulfill its intended purposes (Javdan et al. 2023; Moore et al. 2021). Yet oftentimes the older adults’ needs for the device and the support structure for proper use are overlooked, although these aspects appear crucial for adoption (Moore et al. 2021). Hence, manufacturers and engineers ought to integrate behavioral sciences in the development cycle of their products to design devices that effectively accomplish their expected tasks by taking into account the capabilities of the users and develop the necessary structure (e.g., written or video instructions, help resources) for proper increased adoption and successful implementation (Fisk and Rogers 2002; Javdan et al. 2023), particularly when the intended audience includes older adults (Zhou et al. 2025).

A major technological progress that is pervasively influencing our lives is represented by artificial intelligence (AI, Ferry 2025). Since IBM’s Deep Blue defeat in chess of grandmaster Garry Kimovitch Kasparov in 1997, the ability of computers and computer-controlled robots to perform tasks commonly associated with intelligent beings (a working definition of AI (Copeland 2025)) has not ceased to increase and invade our everyday (digital) lives. Likewise, big data, machine learning, and deep learning algorithms, employed in natural-language processing and large-language models, have undergone dramatic advancements that continuously feed data to train AI systems and produce multimodal (e.g., text, image, audio, video) information. In many fields, especially medicine and linguistics, AI systems are profitably applied to ameliorate human’s capacity to recognize textual and visual patterns (such as when examining clinical psychiatric vignettes, Franco D’Souza et al. (2023), creating radiology workflows, Mese et al. (2023), and making radiologic decisions, Rao et al. (2023), performing clinical examinations in obstetrics and gynecology, Li et al. (2023), and translating written texts into different languages, Ferry (2025). AI can also support individualized cognitive training. Some studies report positive outcomes on training effectiveness, especially by increasing motivation and engagement, and offering individualized learning programs (Adolphe et al. 2025). Others show that AI not only affects favorably cognitive training but also social and emotional health (Vogan et al. 2020). Undoubtedly, AI is already and will continue to bolster human behavior in countless domains.

In scientific research settings, AI systems are increasingly being used to display, analyze, and code real data, create synthetic data, and write text (Abdurahman et al. 2025). Some scholars also use AI to answer opinion polls (so-called in silico sampling), on the premise that AI systems have been trained with huge amounts of data about human attitudes, beliefs, and behaviors, and thus can synthesize that information more effectively than a human brain possibly could (Boelaert et al. 2025). AI is also used to generate scale items and test scoring methods, particularly in educational settings (Ho 2024) and to perform common statistical analyses such as exploratory factor analysis (Koçak 2025). However, most AI systems are not truly open, meaning that the data they have been fed and the algorithms used during their training but also production phases are not transparent and could possibly be biased. Moreover, often these systems evolve very rapidly, thereby decreasing the validity, reproducibility, and replicability of their results. Hence, their responsible use in research requires following a set of guidelines to ensure that the final product can be considered of high quality besides being innovative (Abdurahman et al. 2025; Boelaert et al. 2025; Ho 2024). In general, research is undergoing a new epistemological reconfiguration combining data-driven and hypothesis-driven sciences. Massive amounts of data can now be displayed and analyzed in ways that were unthinkable a decade ago, giving rise to new, previously elusive knowledge. Yet, such knowledge needs to be validated in its usefulness by substantive experts, rather than enjoy blind acceptance. This results in a shift toward exploratory but also reflexive methodologies that allow discovering emerging patterns that must be carefully examined in their assumptions and possible biases before final acceptance (Bouyousfi and Ouedraogo 2024).

## Responsibilities

In concluding this review, it is worthwhile underscoring that we as psychological aging researchers are privileged observers of phenomena and processes that shape how individuals live their third and fourth phases of the lifespan. As such, we are responsible for not only critically contributing to the cumulative scientific corpora on psychological aging, but also to ensure that this scientific knowledge is effectively communicated to the lay audience and to policy and political decision makers.

### About theories

The viewpoint that aging research is “data rich and theory poor” (Birren and Bengston 1988) is probably less endorsed today compared to decades ago, given the advancement of various theoretical perspectives on aging (Johnson and Mutchler 2014; Liang and Luo 2012). Yet, few scholars would argue that current aging theories are fully satisfying and could not benefit from refinement. Various authors plead for a more explicit and clearer investigation of causality mechanisms. Rather than demoting causality discussions to an explicit taboo, which results in speculative inferences and non-transparent implicit assumptions, behavioral and social scientists ought to be explicit and argue openly about causality mechanisms driving developmental changes (or stability), even in observational data (Grosz et al. 2020; Hernán 2018). This can be achieved directly by resorting to classical experimental designs (as discussed by Freund 2015), fully exploiting data from longitudinal designs (Hamaker 2023), and/or by applying formalized frameworks that explicitly lay out and allow testing expectations about causal inferences (e.g., directed acyclic graphs (DAG), Pearl 2009) and various cognitive-, computational-, mathematical-, Bayesian-, connectionist-, and structural causal-modeling techniques (Baribault and Collins 2025; Biazoli et al. 2024; Cavagnaro et al. 2013; Darby and Sederberg 2022; Dixon 2011; Greene and Rhodes 2022; Stine-Morrow et al. 2024; Zacher 2025).

Because developmental aging theories often relate behaviors, attitudes, and beliefs to the passing of time, it is fundamental that aging researchers be very explicit about the temporal dimension implied (Hopwood et al. 2022; Zaheer et al. 1999). This covers thinking about how quickly or slowly processes are supposed to evolve, whether they should be characterized by group patterns or person-specific trajectories, when they ought to take place, and for how long and at what frequency and rhythm they need to be conceptualized (Hamaker 2023). The precise nature of the theorized change also deserves serious contemplation. A fundamental characteristic to be examined is the shape of change. The default linear shape is often inadequate in describing a change-related process, whether in childhood (Grimm et al. 2011), throughout the entire lifespan (McArdle et al. 2002), or old age (Ghisletta et al. 2020). Other important characteristics include continuity vs. discontinuity and reversibility vs. irreversibility (Luhmann et al. 2014). Such thoughtful reflections foster the clarification of the different time scales at which processes can operate, possibly simultaneously but also in opposite directions (for concrete examples, see (Hamaker 2023)).

Finally, given the importance that various types of reserves and resources accumulated over a lifespan can play in old and very old age, there has been a call for aging researchers to consider midlife as a crucial precursor to aging processes (Infurna et al. 2020; Zacher 2025). Cullati et al. (2018) describe in depth how a theoretical framework, centered on biological, physiological, cognitive, economical, and social reserves that are formed, activated, maintained, and reactivated during the lifespan, can help in understanding vulnerability processes in adulthood and aging. Relatedly, Stern (2009) discusses how brain reserves (interindividual differences in structural or functional cerebral features) and cognitive reserves (interindividual differences in the capacity to process mental tasks) can influence how people cope with incipient neurological pathologies that can lead to dementia. In some social sciences, the concept of social capital represents both formal and informal networks of relationships that can assist in advancing one’s goals as an individual and a member of groups (Halpern 2005). Enriching one’s social capital during midlife can help forge a solid basis to face potential challenges emerging in old age. Midlife is when many vulnerabilities first become apparent and when rates of depression, anxiety, and serious psychological distress are highest and represent adversities likely to carry over into later phases of the lifespan if not adequately faced. A better understanding of midlife processes, or at least their individualized historical accounts, can thus educate many aging phenomena (Infurna et al. 2020; Karel et al. 2012).

### About methods

Methodological developments appear on a daily basis, and it is utterly impossible to digest and master them all. Yet, given the conceptual and methodological challenges mentioned before, studying aging requires continuously familiarizing oneself with new relevant methodologies to at least keep abreast of the current literature. At the same time, some old recommendations remain relevant today. Several scholars urge for designs that go beyond comparisons of extreme age groups (typically younger vs. older adults), considering the many characteristics, besides their chronological age, on which such groups typically differ (Freund and Isaacowitz 2013; Greene and Rhodes 2022; Salthouse 2000; Wohlwill 1970; Zacher 2025). To complement age group comparison designs, one may rely on experiments in which the hypothesized determinant of a formalized causal mechanism is manipulated (induced or simulated) to then assess the consequences on its presumed outcome. Other alternatives consist in employing formalized theories and methods, such as computational and machine-learning models, tested on age-continuous samples where estimated parameters are interpretable in terms of the hypothesized underlying process. Examples include structural equation models that recursively find subgroups with similar patterns (Brandmaier et al. 2016), cognitive models that formalize how latent processes map onto observed behavior (Greene and Rhodes 2022), and novel interpretation techniques that help understanding how random forests and neural networks make predictions about complex psychological relationships (Henninger et al. 2025).

It is widely accepted that longitudinal designs offer unique advantages when studying time-related (stability and change) phenomena (Baltes and Nesselroade 1979; Hofer and Sliwinski 2006; Raz and Lindenberger 2011; Schaie 2005). Yet, as alluded to before, longitudinal studies also present potential confounding effects (Salthouse 2000): time of measurement and cohort effects may bias estimates attributable to age (Schaie 1986), retest effects may influence long-term decline estimates (Rabbitt et al. 2001), and longitudinal samples tend to become increasingly positively selective, compared to the baseline sample, as the study progresses (Little et al. 2000). Methodological and statistical attempts to correct for these confounding effects have been applied successfully (Ferrer and Ghisletta 2011) and thus should be part of the basic toolbox of researchers relying on longitudinal panel-like studies. Collaborative efforts, via integrated data sets and possibly harmonized measures, can further increase the robustness of results obtained from many different longitudinal studies (Hofer and Piccinin 2010; Walhovd et al. 2018; Zuber et al. 2023) and overcome intrinsic dependencies of multiple results stemming from few, sometimes overused data sets (Mroczek et al. 2022).

Statistical methods have been proposed to disentangle within-person from between-person effects in multilevel and structural equation models (two frameworks very frequently used to analyze longitudinal panel data; (Curran and Bauer 2011)). This distinction is very important because time-related processes about a given psychological construct may operate (possibly antagonistically) at different time scales, and lack of both theoretical and methodological clarity in this regard may lead to seemingly implausible results (Voelkle et al. 2018; Zaheer et al. 1999; Zyphur et al. 2020). Although intuitively we might think that within-person mechanisms operate on short-term time scales and between-person processes unfold over longer periods of time, this premise ought to be debated theoretically and tested empirically (Hamaker 2023). Pushing this distinction even further, formal mathematical modeling of complex systems allows shedding light on aging mechanisms that interact with each other nonlinearly in feedback loops (Cohen and Olde Rikkert 2024). Complex system theory has been successfully applied to study how intrinsic capacities (operationalized as a network of muscle strength, walking speed, and chair rises, cognitive performance, depressive symptoms and coping abilities, and hearing and visual acuity) build resilience (i.e., the network becomes denser) to functional decline in older adults (Koivunen et al. 2024) and to discuss how the functional aging system (defined as the dynamic interactions between physical, psychological, cognitive, and social functioning and behavioral factors) impacts quality of life, longevity, and success in prevention and treatment in older adults (Kok et al. 2025).

Finally, coming back to open science, it is now recognized that striving to obtain robust (reproducible and replicable) results obtained in all transparency is a necessary goal that aging researchers should also pursue (Hill and Stine-Morrow 2022; Pruchno et al. 2015; Stine-Morrow 2022). Even when specific ethical considerations may surface (e.g., lack of consent to release participants’ data in longitudinal studies), reliable and feasible alternatives to promote open science practices exist (e.g., creating and sharing synthetic data that are different from the original data but reproduce, via simulation under a given model, their expectations, Grund et al. 2024). To assist researchers in adopting open science practices, software-based workflows and tutorials with concrete examples are freely available (Peikert et al. 2021). Large-scale reviews have shown that preregistration practices do not result in fewer positive findings or smaller effect sizes, nor do they take longer to publish. To the contrary, preregistered studies more often contain power analyses and rely on bigger sample sizes, although they do not necessarily prevent HARKing (hypothesizing after the results are known; van den Akker et al. 2024). Relatedly, articles with fewer fragile *p*-values (presenting frequentist inferential results with *p*-values between 0.01 and 0.05) have become less frequent since the 2015 seminal publication about the reproducibility crisis by the Open Science Collaboration (2015) and are more frequently cited than those with more fragile *p*-values (Bogdan 2025).

### About communication

Despite much research showing that old age can also be characterized by positive views, very often the general public’s perception of that phase of the lifespan remains quite negative (Levy 2017). As Diehl et al. (2020) discuss, this negative view of aging not only can create intergenerational conflicts (as shown recently with the COVID-19 pandemic) but may also prevent older individuals from optimizing their chances for healthy and productive aging. As professionals studying aging mechanisms and processes, we ought to rely on our science and contribute to a new, more nuanced and fair narrative on aging. Indeed, in many countries, most adults aged 65 years or older are in good health (Williamson and Christie 2009), several mental health issues are highest in midlife (Infurna et al. 2020), and there is considerable evidence demonstrating various forms of plasticity also in old age (Hertzog et al. 2008; Lindenberger 2014; Lövdén et al. 2010), confirming the hypothesis that cellular aging is not preprogrammed (Kirkwood 2005). Economic realities depict a more refined picture of old age, too. Older adults are estimated to contribute massively to the economy, by working, but especially informally, via volunteering and caregiving (Carmichael and Ercolani 2014; Gonzales et al. 2015). Increase in health care costs is driven especially by children, rather than by older adults (Cosandey and Taboada 2024), and the highest end-of-life health costs are incurred by adults below 65 years of age suffering from colorectal, breast, and prostate cancers (Panczak et al. 2017).

To be effective, this new narrative on aging ought to be propagated well beyond our daily scientific endeavors, to ensure that the opportunities of psychological aging research are well received at important social, policy, and political levels (Diehl et al. 2020). As researchers, we need to popularize our work to the media and warrant that a lucid and unbiased account of aging is spread. We should also take advantage of various administrative duties (such as university committees) to explain to colleagues (both within and outside of our department) how our research can contribute to the quality of life of individuals (Bertenthal 2002). Such lobbying efforts may promote the incorporation of aging into generalist academic curricula and increase funding allocated by our institutions to advance the field of psychological aging.

## Conclusions

Psychological aging research is of critical importance. As the global population continues to age, this demographic shift is gradually reshaping intergenerational relationships, as well as societal, economic, and political dynamics. Aging is a deeply individualized process, characterized by significant heterogeneity at the population level, which makes generalizations both difficult and potentially misleading. While some older adults may contend with chronic illnesses and shift their goals toward meeting basic needs, others continue to lead meaningful and dignified lives, enriched by new experiences. The complexities inherent in studying psychological aging must not deter us from advancing and communicating our understanding, nor absolve us of the responsibility to do so.

## Data Availability

No datasets were generated or analyzed during the current study.
